# Nonlocal total variation based on symmetric Kullback-Leibler divergence for the ultrasound image despeckling

**DOI:** 10.1186/s12880-017-0231-7

**Published:** 2017-11-28

**Authors:** Shujun Liang, Feng Yang, Tiexiang Wen, Zhewei Yao, Qinghua Huang, Chengke Ye

**Affiliations:** 1 Guangdong Provincial Key Laboratory of Medical Image Processing, School of Biomedical Engineering, Southern Medical University, Guangzhou, 510515 People’s Republic of China; 2 College of Information Engineering, Shenzhen University, Shenzhen, 518060 People’s Republic of China; 3 Shenzhen Institutes of Advanced Technology, Chinese Academy of Sciences, Shenzhen, 518055 People’s Republic of China; 4 School of Electronic and Information Engineering, South China University of Technology, Guangzhou, 510641 People’s Republic of China

**Keywords:** Speckle reduction, Spatiogram, Kullback-Leibler (KL) divergence, Nonlocal total variation, Ultrasound image

## Abstract

**Background:**

Ultrasound imaging is safer than other imaging modalities, because it is noninvasive and nonradiative. Speckle noise degrades the quality of ultrasound images and has negative effects on visual perception and diagnostic operations.

**Methods:**

In this paper, a nonlocal total variation (NLTV) method for ultrasonic speckle reduction is proposed. A spatiogram similarity measurement is introduced for the similarity calculation between image patches. It is based on symmetric Kullback-Leibler (KL) divergence and signal-dependent speckle model for log-compressed ultrasound images. Each patch is regarded as a spatiogram, and the spatial distribution of each bin of the spatiogram is regarded as a weighted Gamma distribution. The similarity between the corresponding bins of the two spatiograms is computed by the symmetric KL divergence. The Split-Bregman fast algorithm is then used to solve the adapted NLTV object function. Kolmogorov-Smirnov (KS) test is performed on synthetic noisy images and real ultrasound images.

**Results:**

We validate our method on synthetic noisy images and clinical ultrasound images. Three measures are adopted for the quantitative evaluation of the despeckling performance: the signal-to-noise ratio (SNR), structural similarity index (SSIM), and natural image quality evaluator (NIQE). For synthetic noisy images, when the noise level increases, the proposed algorithm achieves slightly higher SNRS than that of the other two algorithms, and the SSIMS yielded by the proposed algorithm is obviously higher than that of the other two algorithms. For liver, IVUS and 3DUS images, the NIQE values are 8.25, 6.42 and 9.01, all of which are higher than that of the other two algorithms.

**Conclusions:**

The results of the experiments over synthetic and real ultrasound images demonstrate that the proposed method outperforms current state-of-the-art despeckling methods with respect to speckle reduction and tissue texture preservation.

## Background

Ultrasound imaging is one of the four modern medical imaging techniques that deliver cross-sectional images of a patient’s anatomy and physiology in real time [1]. The use of ultrasound in the diagnosis and assessment of imaging organs and soft tissue structures, as well as human blood, is well established [2]. This technique is progressively achieving an important role in the assessment and characterization of cardiac imaging because it is noninvasive and has a strong ability to identify biological soft tissues [3]. However, ultrasound imaging generates images with low contrast resolution, because of speckle noise.

The speckle pattern, which contains typical light and dark spots in an image, is generated by the interference effect of the ultrasonic echoes and scattering of randomly distributed structure scatters. In fact, speckle is technically not a noise in the typical engineering sense because its texture often carries useful information on the image. Therefore, distinguishing between speckle generated from tissue and that from the received RF signal is essential [3]. The former refers to the image texture and the latter, the speckle noise. Speckle noise is a form of multiplicative noise subjected to Gamma distribution [4–6]. It is the primary factor that limits the contrast resolution in diagnostic ultrasound imaging, thereby limiting the detectability of small low-contrast lesions and rendering the ultrasound images generally difficult for non-specialist to interpret [3]. Therefore, reducing speckle noise is important for the interpretation and computer-aided analysis of ultrasound images. The aim of despeckling is to eliminate the speckle noise and maintain the image boundaries and image texture.

Decreasing the frequency of the transducer can increase the signal-noise-ratio (SNR) in ultrasound images. However, doing so decreases the resolution of the image, and consequently hinders the capturing of internal details of human organs. Meanwhile, high-resolution ultrasound images usually indicate low SNR, which can be increased by despeckling post processing techniques. One of the classical despeckling methods is the Wiener filter, which is a linear filter based on local statistics [7]. It uses the weighted average calculation of sub-region statistics to estimate the statistical measures over different pixel windows [3]. However, the linear filters have an inherent drawback of over-smoothing anatomical details and transitional boundaries [8]. Thus, in the last two decades, researchers have focused on nonlinear filtering methods to remove speckle noise in ultrasound images.

Similar to ultrasound images, the synthetic aperture radar (SAR) images are usually degraded by multiplicative speckle noise. Some local speckle statistics-based adaptive filters proposed for SAR images are also used in ultrasound images [9–15]. These adaptive filters use local speckle statistics to improve smoothness in homogeneous regions where the speckle is fully developed and reduce smoothness appreciably in the other regions showing the useful details of an anatomical structure [3]. Adaptive filters include the Lee filter [9], Kuan filter [10], Frost filter [11], adaptive weighted median filter [12], speckle reducing bilateral filter [13], maximum likelihood filer [14] and MRGMAP filter [15]. These adaptive filters use different weighting coefficients calculated from the subregion statistics over different pixel windows to preserve important image structures while removing speckle noise. However, although these adaptive filters can retain the boundaries, they cannot eliminate speckle noise effectively and are sensitive to the size of the pixel window. When the window size is large, the image edges are blurred.

Anisotropic diffusion, proposed by [16], is an efficient nonlinear technique that can enhance contrast and reduce noise simultaneously [17–19]. It smooths homogeneous image regions and preserves image edges without requiring any information from the image power spectrum [3]. For example, the speckle-reducing anisotropic diffusion filtering has been proposed to enhance image contrast [3]. Wavelet filtering is another method that exploits the decomposition of an image into the wavelet basis and zeros out the wavelet coefficients to despeckle the image. Speckle-reduction filtering in the wavelet domain is based on the concept of the Daubechies Symlet wavelet and soft-thresholding denoising [3]. It was proposed first by Donoho [20] and investigated further by Zhong and Cherkassky [21] and Gupta et al. [22]. However, although diffusion and wavelet filters can suppress speckle noise considerably, they produce excessive smoothing details, particularly in the image boundaries and texture.

Nonlocal denoising methods that use the similarities among small patches have been reckoned to be an effective way to preserve details while decreasing noise [6, 23, 24]. A simple and classic method is the well-known nonlocal means (NLM) filter [23], which measures the similarities among the patches centered at a given pixel in the image and prevents the smoothing of boundaries by assigning only high weights to pixels with similar local properties. However, it cannot be applied to speckle filtering directly since the speckle model of the ultrasound images is not subjected to the Gaussian distribution. The NLM filter has been adapted to Gamma-distributed speckle pattern by introducing a Bayesian estimator to the weighting function and Rayleigh-distributed speckle pattern through the introduction of maximum-likelihood estimation [25, 26]. These adapted methods demonstrate better results than the original NLM filter. The NLM filter generates better structural preservation than the diffusion filters. However, it also generates excessive smoothing textures. Based on the NLM filter, the nonlocal total variation (NLTV) minimization scheme analyzes the patterns around the pixels and performs pattern matching in the global image [27, 28]. The nonlocal total variation norm processes textures and repetitive structures effectively. However, although the NLTV filter performs well in Gaussian noise reduction and sharp boundaries preservation, it cannot be applied to log-compressed ultrasound images directly, because the speckle is not subjected to the Gaussian distribution. Thus, a Bayesian NLTV speckle filter (BNLTV) has been developed for ultrasound images and thus is capable of improving speckle suppression and edge enhancement, outperforming the adaptive filters, diffusion filters, wavelet filters, nonlocal denoising methods, and original NLTV filter [29]. However, the BNLTV filter over-smooth image texture and introduces some artificial traces, while it eliminates the speckle noise. It cannot determine whether or not the speckle received from RF signal.

Another nonlocal denoising method is the blocking matching 3D (BM3D) filter proposed by Dabov et al. [30]. This method combines nonlocal and transform-domain approaches and presents an effective denoising framework by grouping similar patches into a 3D array. It subsequently filters the 3D array by using sparse representation in the transform domain. Finally, it aggregates multiple estimates at each location. Based on the BM3D filter, the SAR-BM3D filter [31] was proposed for despeckling SAR images. The SAR-BM3D filter is generally used to despeckle breast ultrasound images and exhibited the impressive despeckling ability [32].

In this paper, an adapted NLTV speckle filter is proposed to address the problem on speckle noise. This filter can eliminate the speckle noise while maintaining the edges and image texture. For the improvement of the despeckling performance, spatiogram similarity measurement based on symmetric KL divergence is introduced to the similarity calculation between the image patches. The proposed method is validated by considering both real ultrasound images and synthetic noisy images. Before the experiment, a Kolmogorov-Smirnov (KS) test has been conducted on the experimental images to ensure that the images are subjected to Gamma distribution [5]. For the evaluation of the despeckling performance without the ground truth image, the natural image quality evaluator (NIQE) [33] is introduced. NIQE can predict the quality of distorted images (noise, ringing, blur, or blocking), even without any prior knowledge of the reference images or their distortions. The remainder of this paper is organized as follows: Section 2 provides the proposed NLTV filtering procedure. Section 3 reports the experimental results. Finally, Section 4 presents the conclusions.

## Methods

### NLTV algorithm

Based on nonlocal denoising methods, NLTV denoising is generally designed for the zero mean Gaussian noise. Dong et al. [28] and Wen et al. [29] proposed a nonlocal total variation noise removal model for multiplicative noise. In these models, the noisy image *Y* from a noise-free image *X* can be modeled as follows [28, 29]:

1 $$ Y=X+{X}^{\gamma}\eta, $$

where *η* generally denotes a zero mean Gaussian noise with a variance *σ* ^2^. γ is a factor that relies on ultrasound devices and additional image reconstruction processing, and when γ = 0.5, the formation process for the log-compressed ultrasound image can be approximated [12]. Denoising methods aim to restore *X* or find a good estimate $\widehat{X}$ of *X* from *Y*.

The NLTV denoising method searches similar patches in a certain searching window centered at a given pixel and obtains the weight coefficients according to the similarities among the patches. The restored pixel is reconstructed by minimizing nonlocal TV model. This operation is repeated for each pixel in the sliding searching window fashion in the whole noisy image. The details of the NLTV denoising process are described in the subsequent text.

Given a *p* × *p* patch and a *iw* × *iw* searching window centered at a certain pixel, *i*, in the noisy image, *m* similar patches (including the given patch) are found. The distance of two patches can be formulated as follows [28]:

2 $$ d\left(i,j\right)={G_{\alpha}}^{\ast}\left({\left\Vert X\left(i+\bullet \right)-X\left(j+\bullet \right)\right\Vert}^2\right), $$

where *G*_*α*_ is a Gaussian kernel with standard deviation *α*, X(*i* + ∙) and X(*j* + ∙) are the patches centered at *i* and *j*, respectively, in a given searching window. The similarity coefficients can be calculated as follows [28]:

3 $$ w\left(i,j\right)=\mathit{\exp}\left(\frac{-d\left(i,j\right)}{2{h}^2}\right), $$

where *h* is a filtering parameter. The smaller the distance, the greater the *w*(*i*, *j*), which means the two patches is more similar.

Then the NL gradient is defined based on the weighting of similar patches, which is determined as follows [28]:

4 $$ \left|{\nabla}_{NL}{X}_i\right|=\sqrt{\sum_j{\left(X(j)-X(i)\right)}^2w\left(i,j\right)} $$

Based on the Bayesian rational [29] and Eq. (4), the restored gray value of *i* can be obtained by minimizing the nonlocal TV model represented as follows [28]:

5 $$ \mathit{\min}E\left({X}_i\right)={\sum}_i\left|{\nabla}_{NL}{X}_i\right|+\frac{\lambda }{2}{\sum}_i{\left({X}_i-{Y}_i\right)}^2, $$

where *λ* > 0 and is a Lagrange parameter balancing the influence between the nonlocal regularization term ∑_*i*_|*∇*_*NL*_ *X*_*i*_| and data fidelity term.

The above operation is performed in each pixel in the sliding search window fashion in the whole noisy image. In this operation, an efficient minimizing algorithm is necessary to improve the performance at a relatively fast rate. Many efficient algorithms for the computation of the nonlocal TV problem have been proposed [34, 35]. Of these algorithms, the split Bregman method can solve the nonlocal TV minimization efficiently. It simplifies the nonlocal TV problem to two sub-problems calculated by the Gauss–Seidel iterative scheme and soft-thresholding formula [34].

### Symmetric KL divergence NLTV speckle filter

The NLTV method was originally proposed for additive Gaussian noise removal [27]. It uses the non-robust Euclidean distance to measure the similarities among the patches. To improve the performance of the NLTV despeckling method, we propose to replace the similarity measurement based on Euclidean distance with a spatiogram similarity measurement based on symmetric KL divergence. This similarity measurement is performed on the noisy patches to determine the similarities between each pair of patches. Each patch is regarded as a spatiogram, and the spatial distribution of each bin of a spatiogram is regarded as a weighted Gamma distribution, where the weight is the probability of the corresponding bin; subsequently, the similarity in the corresponding bins of the two spatiograms is computed by the symmetric KL divergence with two weighted Gamma distributions [36].

Given a spatiogram,

6 $$ h=\left\{{n}_b,{\mu}_b,{\Sigma}_b\right\}\kern0.5em \left(b=1,2,\dots, B\right) $$

where *n*_*b*_ is the probability value of the bin, *μ*_*b*_ and Σ_*b*_ are the mean and covariance matrix, respectively, of the pixel coordinates in the bin, *B* is the number of bins. A weighted Gamma distribution is represented as follows:

7 $$ {f}_b(x)={n}_b{p}_b, $$

where

8 $$ {p}_b=\frac{\beta^{\alpha }}{\Gamma \left(\alpha \right)}{x}^{\alpha -1}{e}^{-\beta x}, $$

where α = *μ* ^2^/Σ, β = *μ*/Σ. The KL distance between the two weighted Gamma distributions, *f*_*b*_(*x*) and ${f}_b^{\hbox{'}}(x)$, can be calculated as follows:

9 $$ {d}_{KL}\left({f}_b\mid \left|{f}_b^{\hbox{'}}\right)={d}_{KL}\right({n}_b{p}_b\mid \left|{n}_b^{\hbox{'}}{p}_b^{\hbox{'}}\right)={n}_b{d}_{KL}\Big({p}_b\mid \left|{p}_b^{\hbox{'}}\right)+{n}_b\log \left({n}_b/{n}_b^{\hbox{'}}\right), $$

where

10 $$ {d}_{KL}\operatorname{}{p}_b\mid \left|{p}_b^{\prime}\right)=\mathit{\log}\frac{\beta^{\alpha}\varGamma \left({\alpha}^{\prime}\right)}{{\beta^{\prime}}^{\alpha^{\prime }}\varGamma \left(\alpha \right)}+\left(\alpha -{\alpha}^{\prime}\right)-\left(\beta -{\beta}^{\prime}\right), $$

where *d* is the space dimensionality (for spatiogram, *d* = 2). The symmetric KL distance between the two weighted Gamma distributions is represented as follows:

$$ {d}_{SKL}\left({f}_b,{f}_b^{,}\right)=\frac{d_{KL}\left({f}_b,\mid \mid, {f}_b^{\hbox{'}}\right)+{d}_{KL}\left({f}_b^{\hbox{'}}||{f}_b\right)}{2} $$

11 $$ ={n}_b{d}_{KL}\left({p}_b||{p}_b^{\hbox{'}}\right)/2+{n}_b^{\hbox{'}}{d}_{KL}\left({p}_b^{\hbox{'}}||{p}_b\right)/2+\left({n}_b-{n}_b^{\hbox{'}}\right)\log \left({n}_b-{n}_b^{\hbox{'}}\right)/2. $$

Then, the weight coefficient between the two patches can be calculated as follows:

12 $$ w\left(i,j\right)=\mathit{\exp}\left(\frac{-{\sum}_{b=1}^B{d}_{SKL}\left({f}_b,{f}_b^{\hbox{'}}\right)}{2{h}^2}\right). $$

### Split-Bregman implementation

After obtaining the weight coefficients by Eq. (12), we use the Split Bregman iteration [28] to minimize the adapted NLTV-functional Eq. (5).

To prevent the singularity and complexity of the numerical difference, Goldstein and Osher [35] proposed an alternating minimization scheme. They introduced an auxiliary variable to approximate the image gradient. The NLTV functional can be modified as the following minimization problem by introducing the auxiliary variable *d* instead of *∇*_*NL*_ *X*, such that [28]

13 $$ \mathit{\min}E\left(X,d\right)={\sum}_i\left|d\right|+\frac{\lambda }{2}{\sum}_i{\left(X-Y\right)}^2\kern0.5em s.t.d={\nabla}_{NL}X. $$

The above constrained problem can be converted into an unconstrained problem by using the quadratic penalty method [28].

14 $$ \mathit{\min}E\left(X,d\right)={\sum}_i\left|d\right|+\frac{\beta }{2}{\left(d-{\nabla}_{NL}X\right)}^2+\frac{\lambda }{2}{\sum}_i{\left(X-Y\right)}^2. $$

Then, the Bregman iteration is used to solve the above minimization [28].

15 $$ \left(X,d\right)=\mathit{\min}{\mathit{\arg}}_{X,d}{\sum}_i\left|d\right|+\frac{\lambda }{2}{\sum}_i{\left(X-Y\right)}^2+\frac{\beta }{2}{\sum}_i{\left|d-{\nabla}_{NL}X-b\right|}^2, $$

Where *β* is a positive constant and *b* is the Bregman parameter.

Apparently, Eq. (15) is a multivariate function and can be minimized by alternatively solving the two minimization subproblems with respect to *X* and *d*. Fixed *d*, the minimization over *X* is [28]

16 $$ X=\mathit{\min}{\mathit{\arg}}_X\frac{\lambda }{2}{\sum}_i{\left(X-Y\right)}^2+\frac{\beta }{2}{\sum}_i{\left|d-{\nabla}_{NL}X-b\right|}^2. $$

The optimal solution of *X* satisfies the following equation using the Euler-Lagrange formula [28]:

17 $$ -\beta {\mathit{\operatorname{div}}}_{NL}\left(d-{\nabla}_{NL}X-b\right)-\lambda \left(X-Y\right)=0 $$

To get a fast solution for Eq. (17), we use the Gaussian-Seidel iteration, and the solution is represented as [28]

18 $$ \widehat{X_i^{k+1}}=\frac{1}{\lambda +\beta {\sum}_j{w}_{ij}}\left(\beta {\sum}_j{w}_{ij}{X}_j^k+\lambda {Y}_i-\beta {\sum}_j\sqrt{w_{ij}}\left({d}_{ij}^k-{b}_{ij}^k-{d}_{ji}^k+{b}_{ji}^k\right)\right). $$

Fixed *X*, the minimization over *d* is [28]

19 $$ d=\mathit{\min}{\mathit{\arg}}_d{\sum}_i\left|d\right|+\frac{\beta }{2}{\sum}_i{\left|d-{\nabla}_{NL}X-b\right|}^2. $$

According to the soft-thresholding formula [28], the solution of Eq. (19) is provided by

20 $$ {d}^{k+1}=\frac{\nabla_{NL}{X}^{k+1}+{b}^k}{\left|{\nabla}_{NL}{X}^{k+1}+{b}^k\right|}\cdotp \mathit{\max}\left(\left|{\nabla}_{NL}{X}^{k+1}+{b}^k\right|-\frac{1}{\beta },0\right). $$

Finally, the Bregman variable *b* is updated as follows [28]:

21 $$ {b}^{k+1}={b}^k+{\nabla}_{NL}{X}^{k+1}-{d}^{k+1}. $$

The algorithm of NLTV model is summarized in Algorithm 1. The procedure consists of two steps: the NL weights computation step and NLTV minimization step. In the NL weights computation step, the KL distance is calculated using Eq. (11). It is then used to calculate the NL weights using Eq. (12). After the NL weights are calculated, the NLTV minimization step is performed using the Split-Bregman scheme. In this algorithm, *n* represents the number of Gaussian-Seidel iteration and is used to determine a good approximation in Eq. (18). Meanwhile, *k* is the number of the overall iteration.
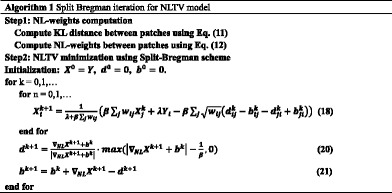


## Experiments

The synthetic noisy images and clinical ultrasound images from [29] are studied to assess and compare the performances of the despeckling methods quantitatively. In [29], a 2D synthetic image “Modified Shepp-Logan Phantom” available in MATLAB is considered and corrupted with different noise levels. The speckle simulation for the synthetic image is based on the noisy model, the Eq. (1). Three levels of noise are tested by setting standard deviations  *sigma* = {0.8,1.0,1.3}.The clinical ultrasound images include liver, intravascular, and 3D ultrasound images. Many experiments performed on log-compressed ultrasound images showed that the speckle noise is better described by the Gamma distribution [4–6, 37]. A simple experiment is then conducted to determine whether or not the speckle noise is subjected to Gamma distribution. In this experiment, the Kolmogorov-Smirnov (KS) test is performed on the experimental images [5]. For each image, the comparison between Gaussian, Gamma, and Rayleigh distribution is done. The formula of the KS test is as follows [5]:

22 $$ {D}_{KS}=\mathit{\sup}\left|\widehat{F_n}(i)-{F}_X(i)\right|, $$

where $\widehat{F_n}$ is the empirical cumulative distribution function (CDF) of the observed images, *F*_*X*_ is the CDF of either Gaussian, Gamma, − Rayleigh, or Fisher-Tippett distribution. The Glivenko-Gantelli theorem states that, if the samples are subjected to distribution *F*_*X*_, then *D*_*KS*_ converges to 0 [38]. In Table 1, the values in the Gamma column are smaller than the other values, and this means the real ultrasound images and synthetic noisy image are tended to subject to the Gamma distribution.

**Table 1:** The results of the KS test on the clinical ultrasound images and synthetic noisy images

Distribution	Gaussian	Rayleigh	Fisher-Tippett	Gamma
Shepp-Logan Phantom Gamma-distributed	0.5699	0.9884	0.3871	0.2210
Field II Kidney Phantom	0.3812	0.9673	0.3751	0.0781
Liver	0.3451	0.9567	0.3761	0.1261
IVUS	0.4523	0.9907	0.3744	0.0270
3DUS	0.3432	0.9549	0.3716	0.1195

Three measures are adopted for the quantitative evaluation of the despeckling performance: the signal-to-noise ratio (SNR), structural similarity index (SSIM), and natural image quality evaluator (NIQE). The SNR is established on the variance ratio of the effective signal and noise using the despeckled and noise-free image such that

23 $$ SNR=20{\mathit{\log}}_{10}\frac{\sqrt{\frac{1}{M}{\sum}_i^M{X}_i^2}}{\sqrt{\frac{1}{M}{\sum}_i^M{\left(\widehat{X_i}-{X}_i\right)}^2}}, $$

where *M* is the total number of pixels in the noisy image, $\widehat{X_i}$ and *X*_*i*_ are the restored value and true value at pixel *i*, respectively. The SSIM is used to measure the structural similarity between the images [39, 40], and is defined as follows:

24 $$ SSIM=\frac{\left(2{\mu}_X{\mu}_{\widehat{X}}+{c}_1\right)\left(2{\sigma}_{X\widehat{X}}+{c}_2\right)}{\left({\mu}_X^2+{\mu}_{\widehat{X}}^2+{c}_1\right)\left({\sigma}_X^2+{\sigma}_{\widehat{X}}^2+{c}_2\right)}, $$

where *μ*_*X*_ and ${\mu}_{\widehat{X}}$ are the mean of the ground truth *X* and denoised image $\widehat{X}$, respectively, ${\sigma}_{X\widehat{X}}$ is the covariance of *X* and $\widehat{X}$, and *c*_1_ and *c*_2_ are constant parameters. In this research, the SSIM is locally measured with an 8 × 8 Gaussian kernel, and the mean SSIM (MSSIM) is estimated as a global SSIM by all the local SSIMs. The NIQE [33] is based on the construction of a “quality aware” collection of statistical features, which are based on a simple and successful space domain natural scene statistic model, represented as

25 $$ \mathrm{D}=\sqrt{{\left({v}_1-{v}_2\right)}^T{\left(\frac{\varSigma_1+{\varSigma}_2}{2}\right)}^{-1}\left({v}_1-{v}_2\right),} $$

where *v*_1_, *v*_2_ and Σ_1_, Σ_2_ are the mean vectors and covariance matrices, respectively, of the natural Multivariate Gaussian (MVG) model and MVG model of a distorted image.

The proposed algorithm is compared with two adaptive algorithms, namely, the BNLTV filter [29], which is based on Bayesian framework, and SAR-BM3D filter [31] to provide relevant comparisons. The results of the [29] are provided by the author. The parameters of proposed filter and SAR-BM3D filter are adjusted to obtain the best SNR scores. The detailed parameters for the proposed filter and SAR-BM3D filter are listed in Table 2. All the algorithms are implemented with MATLAB R2012a, and the computer is equipped with 2.80 GHz CPU (10-core, E5) and 64 GB RAM.

**Table 2:** Parameters Settings for the Proposed Algorithms

Data Set Algorithm			Shepp_Logan Phantom sigma = {0.8;1.0;1.3}	Field II Kidney Phantom	Liver	IVUS	3DUS
SAR-BM3D	Number of looks	*L*	1	1	1	1	1
	Patch Size	*N*_*i*_	8 × 8	8 × 8	8 × 8	8 × 8	8 × 8
	Search_Window	*|△*_*i*_ *|*	11 × 11	11 × 11	11 × 11	11 × 11	11 × 11
Proposed	Lambda	*λ*	{30;35;20}	30	30	30	30
	Beta	*β*	{25;25;25}	25	25	25	25
	Bandwidth	*h*	{0.2;0.2;0.2}	0.3	0.3	0.5	0.3
	Search_Window	*|△*_*i*_ *|*	{31 × 31;41 × 41;41 × 41}	31 × 31	11 × 11	11 × 11	11 × 11
	Patch_Size	*N*_*i*_	5 × 5	5 × 5	7 × 7	7 × 7	7 × 7
	Inner_Iteration	*n*	2	2	2	2	2
	Outer_Iteration	*k*	50	50	50	50	50

### Filter parameters

**Fig. 1:**
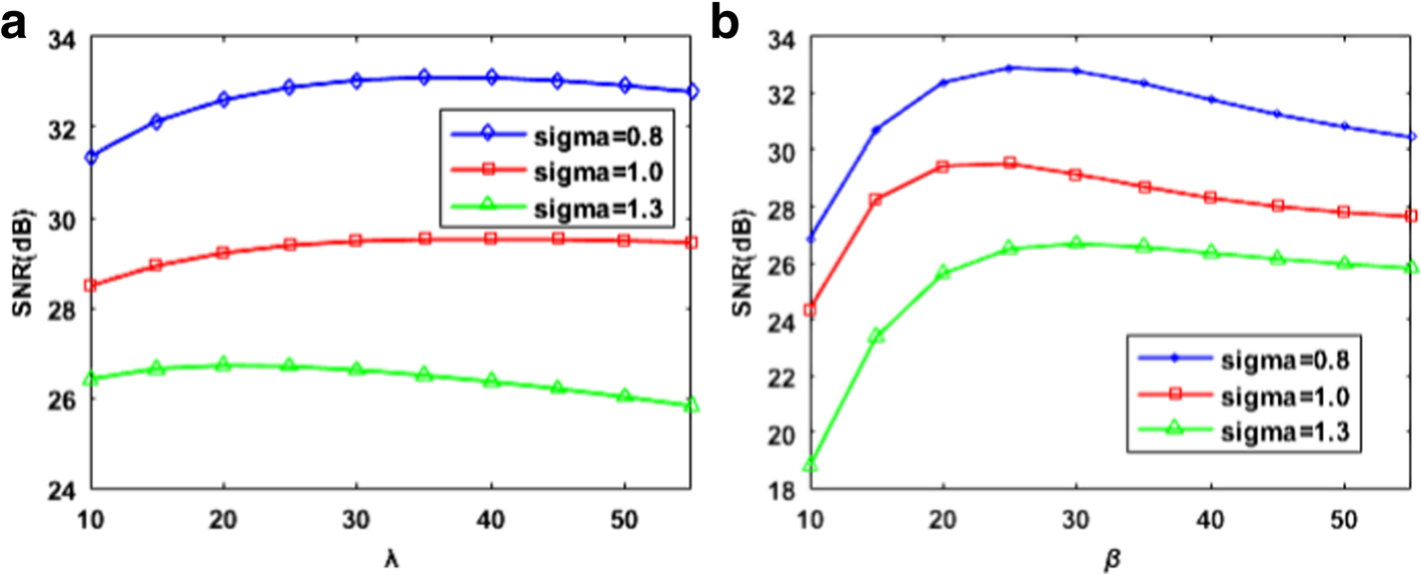
The impact of nonlocal total variation scheme-related parameters for the proposed method. **a**The signal-to-noise ratio (SNR) as a function of lambda *λ*. **b** The signal-to-noise ratio (SNR) as a function of beta *β*

The performance of the proposed despeckled method depends on the setting of the two patch-related parameters (i.e., the patch size *p* and the number of similar patches in a search window *m*) and two nonlocal total variation scheme-related parameters. The parameters are the Lagrange parameter *λ*, which balances the action between the nonlocal regularization and data fidelity terms, and the quadratic penalty parameter *β*, which must be large enough to ensure *d* is sufficiently close to *∇*_*NL*_ *X*. In our despeckling experiments with synthetic noisy images, *p* and *m* are selected as 5 and 10, respectively, which yield higher denoising SNR. For the real ultrasound images, the proposed algorithm is implemented with parameters of *p* and *m* with values of 7 and 10, respectively. The suitable values for *λ* and *β* are then ascertained by implementing the proposed algorithm on the synthetic noisy images with varying *λ* and *β* values. The SNR values are used as quantitative indicators to evaluate the sensitivity to the setting of these two parameters. For the proposed algorithm, the curves as a function of the *λ* and *β* parameters are shown in Fig. 1. These two parameters have an apparent effect on the speckle suppression and edge sharpness of the despeckling image. As shown in Fig. 1, the speckle cannot be reduced because of the small Lagrange parameter λ and quadratic penalty parameter *β*. Meanwhile, large *λ* and *β* values result in a blurred edge transition region. Only the proper parameters can balance the performance between speckle reduction and boundary preservation. Based on the observation from Fig. 1, λ and β are fixed at 30 and 25 in the following experiments while using the clinical ultrasound images, respectively, which yield an improved SNR for data with varying noise levels, although these two values are not strictly optimal for each image.

**Fig. 2:**
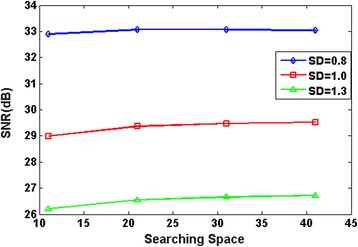
The impact of the searching space *|Δ*_*i*_ *|* for the proposed method

The searching window size is another parameter that affects the despeckling effect. The effect of searching window size is illustrated in Fig. 2, where the experimental results demonstrate that larger search windows do not necessarily produce favorable results. When the searching window size increases, the searching time increases dramatically, whereas the SNR score has no significant difference. The result implies that a 40 × 40 searching window size is suitable for despeckling and can reduce the computation time.

## Results

### Despeckling of synthetic noisy images

The SNR and MSSIM of the BNLTV, SAR-BM3D, and proposed methods on the Shepp–Logan phantom image corrupted with Gamma-distributed noise are shown in Tables 3 and 4. With regard to SNR, the proposed method significantly outperforms the other two methods in images under all noise levels (standard deviation (sigma) from 0.8 to 1.3). When the noise level increases, the proposed algorithm achieves slightly higher SNRS than the SAR-BM3D algorithm, and the SSIMS yielded by the proposed algorithm is obviously higher than that of the SAR-BM3D algorithm. The proposed algorithm outperforms SAR-BM3D algorithm with SNR improvements ranging from 2.02 dB to 4.51 dB and SSIM improvements from 0.0036 to 0.0063. Compared with the BNLTV algorithm, SNR improvements of the proposed method are ranging from 0.54 dB to 4.93 dB and SSIM improvements from 0.0041 to 0.005.

**Table 3:** The SNR Comparison under Different Gamma distribution Conditions

	BNLTV	SAR-BM3D	Proposed
sigma = 0.8	28.11	28.53	33.04
sigma = 1.0	26.92	26.78	29.52
sigma = 1.3	26.18	24.7	26.72

**Table 4:** The MSSIM Comparison under Different Gamma distribution Conditions

	BNLTV	SAR-BM3D	Proposed
sigma = 0.8	0.9921	0.9921	0.9962
sigma = 1.0	0.9903	0.9917	0.9953
sigma = 1.3	0.99	0.9891	0.9945

**Fig. 3:**
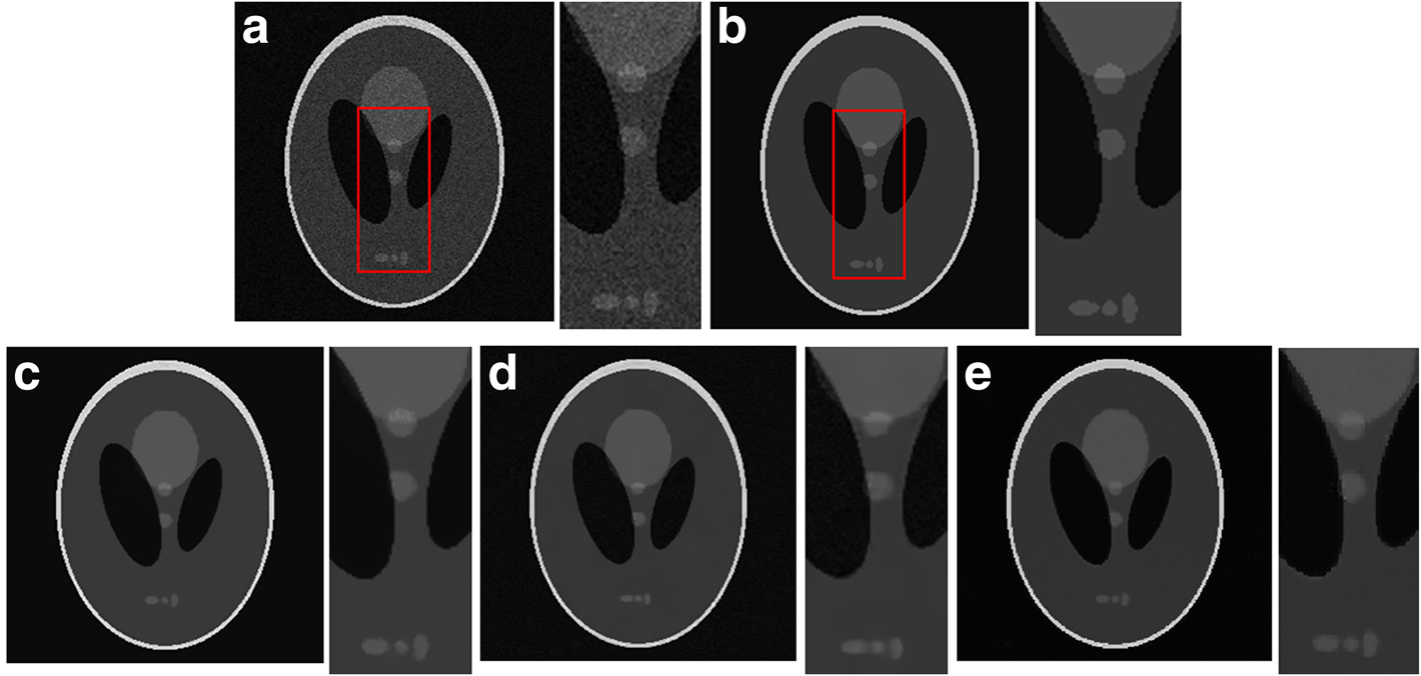
Results obtained with the compared filters applied to the Shepp-Logan phantom image corrupted with Gamma-distribution noise (standard deviation (sigma) = 1.3). **a**The noisy image and its corresponding enlarged representative region labeled with the red rectangle. **b**The ideal image and its corresponding representative region. (c)-(e)Outputs of the compared filters and the same representative regions corresponding to the filtered image, **c** BNLTV, **d** SAR-BM3D, **e** The proposed method. BNLTV = Bayesian NLTV; SAR-BM3D = blocking matching 3D filter of SAR image

Figure 3 provides a visual evaluation of the despeckling results from the Shepp–Logan phantom image corrupted with Gamma-distributed noise corresponding to sigma of 1.3. The proposed algorithm shows better performance in removes speckle without considerable detail loss and edge blurring and thus exhibits better performance than the other despeckling algorithm. The BNLTV result has the edge preservation but with the texture information loss. The proposed method generates fewer intensity oscillations in the homogeneous region than the SAR-BM3D method.

**Fig. 4:**
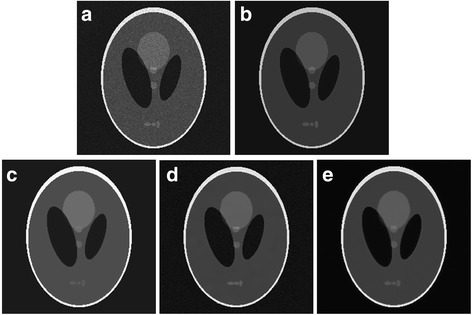
Results obtained with the compared filters applied to the Shepp-Logan phantom image corrupted with Rayleigh-distribution noise (standard deviation (sigma) = 1.3). **a** The noisy image and its corresponding enlarged representative region labeled with the red rectangle. **b** The ideal image. **c-e** Outputs of the compared filters, **c** BNLTV, **d** SAR-BM3D, **e** The proposed method. BNLTV = Bayesian NLTV; SAR-BM3D = blocking matching 3D filter of SAR image

The Shepp–Logan phantom image corrupted by the Rayleigh-distributed speckle with sigma of 1.3 is used to evaluate the ability of the despeckling filters to manage speckles with different distributions. The results obtained from the SAR-BM3D, BNLTV and proposed method on the Rayleigh corrupted image are shown in Fig. 4. Table 5 presents the SNR and MSSIM scores in despeckling the Rayleigh corrupted image. Among the methods, the proposed method has the highest SNR and MSSIM scores for the Rayleigh-based image.

**Table 5:** The SNR and MSSIM Indices for the Shepp-Logan Phantom under Rayleigh distribution Conditions with sigma = 1.3

	BNLTV	SAR-BM3D	Proposed
SNR	19.88	31.541	36.8
MSSIM	0.9550	0.9939	0.9987

### Despeckling of field II kidney phantom noisy image

**Fig. 5:**
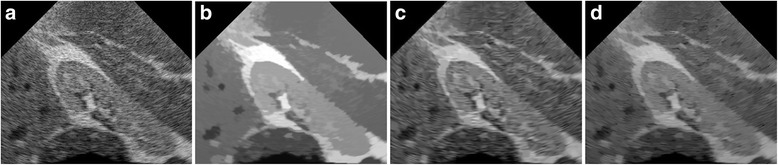
Results obtained with the compared filters for the Field II simulated B-mode image of synthetic kidney. **a** The synthetic kidney image. **b-d** Outputs of the compared filters, **b** BNLTV **c** SAR-BM3D, **d** The proposed method. BNLTV = Bayesian NLTV; SAR-BM3D = blocking matching 3D filter of SAR image

The performance of the proposed algorithm on the image is verified by performing Field II simulation of speckle noise. The B-mode image of a synthetic kidney is shown in Fig. 5a. The results despeckled by the three methods are given in the Fig. 5b–d. All the algorithms exhibit improved speckle noise reduction performance. Notably, the result of the BNLTV filter has the most texture information loss and artificial traces compared with the other two despeckling methods. Compared with the SAR-BM3D filter, the proposed method shows stronger speckle noise reduction ability.

As no ground truth image for the simulated kidney image is present, a blind or no-reference image quality assessment metric, natural image quality evaluator (NIQE), is introduced. The quantitative results are listed in Table 6. The results show that the proposed method achieves the highest NIQE value, because it can maintain the texture of the image while removing the speckle noise.

**Table 6:** The NIQE values on the Synthetic Kidney Phantom Images

	BNLTV	SAR-BM3D	Proposed
Kidney Phantom	6.04	6.71	7.96

### Despeckling of clinical ultrasound image

The effectiveness of the proposed approach on the real ultrasound images is verified. The experiments are conducted on real 2D liver ultrasound image, intravascular ultrasound (IVUS) image, and 3D ultrasound image. Compared with the previous synthetic images, these clinical images contain more fruitful structure information, such as the edges, tiny features, and uniform areas.

**Fig. 6:**
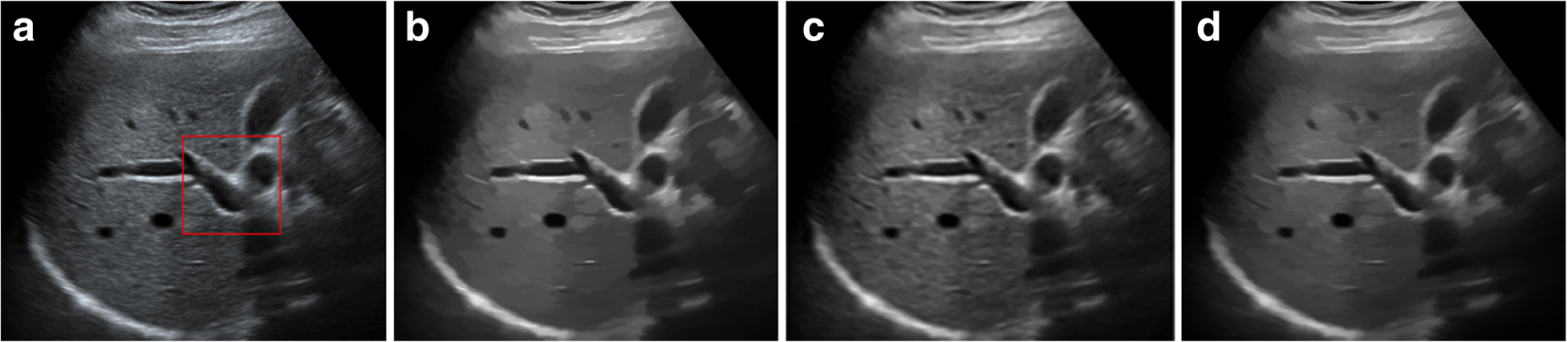
Results obtained with compared filters applied to the liver ultrasound image. **a** The original image. **b-d** Outputs of the compared filters, **b** BNLTV, **c** SAR-BM3D, **d** The proposed method. BNLTV = Bayesian NLTV; SAR-BM3D = blocking matching 3D filter of SAR image

**Fig. 7:**
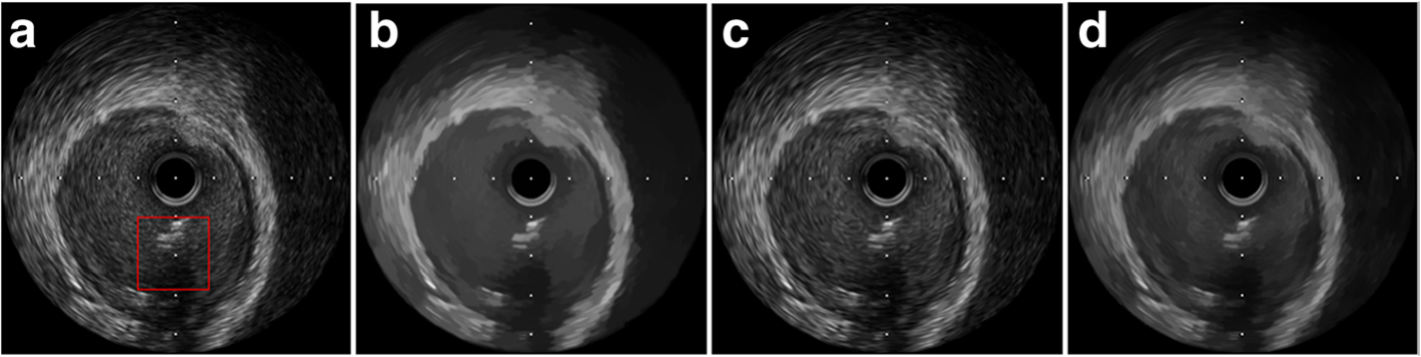
Results obtained with the compared filters applied to the IVUS image. **a** The original image. **b-d** Outputs of the compared filters, **b** BNLTV, **c** SAR-BM3D, **d** The proposed method. BNLTV = Bayesian NLTV; SAR-BM3D = blocking matching 3D filter of SAR image

**Fig. 8:**
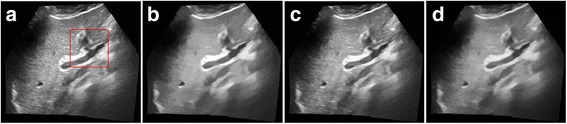
Results obtained with the compared filters applied to the 3D ultrasound image. **a** The original image. **b-d** Outputs of the compared filters, **b** BNLTV, **c** SAR-BM3D, **d** The proposed method. BNLTV = Bayesian NLTV; SAR-BM3D = blocking matching 3D filter of SAR image

**Fig. 9:**
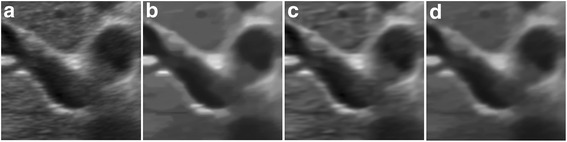
The representative regions corresponding to the red rectangle region in Fig. 6a for the liver ultrasound image. **a**The original image. **b-d** Outputs of the compared filters, **b** BNLTV **c** SAR-BM3D, **d** The proposed method. BNLTV = Bayesian NLTV; SAR-BM3D = blocking matching 3D filter of SAR image

**Fig. 10:**
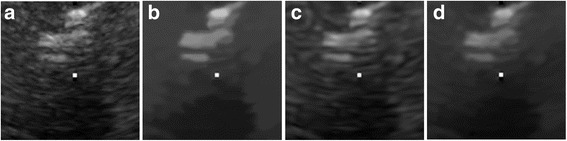
The representative regions corresponding to the red rectangle region in Fig. 7a for the IVUS image. **a** The original image. **b-d** Outputs of the compared filters, **b** BNLTV, **c** SAR-BM3D, **d** The proposed method. BNLTV = Bayesian NLTV; SAR-BM3D = blocking matching 3D filter of SAR image

**Fig. 11:**
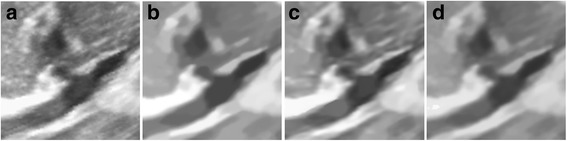
The representative regions corresponding to the red rectangle region in Fig. 8a for the 3D ultrasound image. **a** The original image. **b-d** Outputs of the compared filters, **b** BNLTV, **c** SAR-BM3D, **d** The proposed method. BNLTV = Bayesian NLTV; SAR-BM3D = blocking matching 3D filter of SAR image

The despeckled results of these compared methods on the real 2D liver ultrasound, IVUS, and 3D ultrasound images are presented in Figs. 6, 7, and 8, respectively. The proposed method reduces the speckle noise considerably without losing substantial texture information and smooths the details. As shown by the enlarged images in Figs. 9, 10, and 11, the BNLTV filter slightly smooths the image texture information slightly. Notably, the proposed filter generates the most visually pleasant results. These observations are consistent with the results obtained from the synthetic data.

The quantitative perceptual results of NIQE scoring are provided in Table 7. The results show that the proposed method has the highest NIQE values for 2D liver, IVUS and 3D ultrasound images.

**Table 7:** The NIQE values on the Liver, IVUS and 3DUS Images

	BNLTV	SAR-BM3D	Proposed
Liver	6.86	7.58	8.25
IVUS	5.49	6.06	6.42
3DUS	8.33	7.27	9.01

## Discussion

The excellent despeckling performance of the proposed filter can be attributed to the spatiogram similarity based on symmetric KL divergence. This similarity measurement is more adaptable to the complex speckle noise, because the KL divergence can measure the difference between two probability distributions and the spatiogram can consider the spatial information of the image patches. Therefore, it demonstrates better performance than the similarity computation based on the non-robust Euclidean distance, because Euclidean distance uses only the intensity information of the pixels in the patch.

Finally, the limitation of the proposed filter is that its filtering parameters (*m*, *p*, *λ*, and *β*) are currently manually determined by experience. The optimal parameter setting may change with noise levels and image characteristics. Although the experimental results in this study show that the proposed filter can achieve state-of-the-art performance with experiential parameters, the automatic determination of filtering parameters with theoretical foundations is warranted in a future study.

## Conclusion

In this paper, an adapted NLTV-based speckle filter has been presented to despeckle the ultrasound images. The filter exploits the spatiogram similarity based on the symmetric KL divergence for the similarity calculation between image patches. As a result, the performance of NLTV is improved. The adapted filter is applied on synthetic and real ultrasound images and then compared with current state-of-the-art methods. The results demonstrate that the proposed filter outperforms current state-of-the-art filters with regard to ultrasound despeckling. We believe that our method can improve the quality of ultrasound images and has benefit effects on visual perception and diagnostic operations.

## Abbreviations

BM3D: Blocking matching 3D filter
BNLTV: Bayesian NLTV speckle filter
KL: Kullback-leibler
KS test: Kolmogorov-smirnov test
NIQE: Natural image quality evaluator
NLM: Nonlocal means
NLTV: Nonlocal total variation
SAR: Synthetic aperture radar
SNR: Signal-to noise ratio
SSIM: Structural similarity index
